# Downregulation of selenium-binding protein 1 is associated with poor prognosis in lung squamous cell carcinoma

**DOI:** 10.1186/s12957-016-0832-6

**Published:** 2016-03-08

**Authors:** Xing Tan, Li Liao, Yan-Ping Wan, Mei-Xiang Li, Si-Han Chen, Wen-Juan Mo, Qiong-Lan Zhao, Li-Fang Huang, Gu-Qing Zeng

**Affiliations:** School of Nursing, University of South China, 28# Changsheng Road West, Hengyang, 421001 Hunan China; School of Medicine, University of South China, Hengyang, 421001 China

**Keywords:** Selenium-binding protein 1, Lung squamous cell carcinoma, Prognosis

## Abstract

**Background:**

We found that selenium-binding protein 1 (SBP1) was progressively decreased in the human bronchial epithelial carcinogenic processes. Knockdown of SBP1 in immortalized human bronchial epithelial cell line 16HBE cells significantly increased the efficiency of B[a]P-induced cell transformation. However, the relationship between SBP1 expression and clinicopathological factors of patients has not been defined completely. The specific role of SBP1 in prognosis of lung squamous cell carcinoma (LSCC) is still unknown.

**Methods:**

Tissue samples from 82 patients treated by pulmonary lobectomy for LSCC were used. Immunohistochemistry and western blotting were used to detect the expressions of SBP1 protein. The relationships between the expression level of SBP1 and the clinicopathological features of patients were analyzed. Cox proportional hazard regression analysis and Kaplan–Meier method were used to perform survival analysis.

**Results:**

Expressions of SBP1 proteins were significantly lower in LSCC tissues than that in the corresponding normal bronchial epithelium (NBE) tissues (*P* = 0.000). In LSCC, The expression levels of SBP1 had not correlated with patients’ age, gender, smoking state, primary tumor stages (T), TNM clinical stages, and distant metastasis (M) (*P* > 0.05). However, downregulation of SBP1 was significantly associated with higher lymph node metastasis and lower overall survival rate (*P* < 0.05). Cox regression analysis indicated low expressions of SBP1 can be an independent prognostic factor for poor overall survival in LSCC patients (*P* = 0.002).

**Conclusions:**

Downregulation of SBP1 may play a key role in the tumorigenic process of LSCC. SBP1 may be a novel potential prognostic factor of LSCC.

## Background

In the global scale, lung cancer is one of all common carcinoma, which always keeps the leading position, and is the first cancer in the morbidity and mortality of carcinoma. According to the GLOBOCAN 2008 data, there are 23 % of total cancer-related mortalities and 17 % of newly diagnosed cancer cases for primary lung cancer [1]. Over the past 30 years in China, the mortality rate of lung cancer has increased by 465 % [2], it is responsible for more deaths than prostate, colon, and breast tumors combined [3]. Although there are great advances recently in the cancer treatments, the prognosis of patients with lung cancer is poor even after curative surgery and chemotherapy, the rate of 5-year survival is less than 15 % [4]. The main reasons for the low survival rate of the patients could involve the lack of sensitive and specific biomarkers for prognosis of lung cancer. Therefore, it is necessary to identify the biomarkers for the prognosis of lung cancer that lead to enhancing more effective individual therapies, reduce the mortality, and increase 5-year survival rate.

Classically, lung cancer is pathologically classified into non-small cell lung cancer (NSCLC) and small cell lung cancer (SCLC) [5]. NSCLC is divided into the several histologic subtypes: squamous cell carcinoma (SCC), adenocarcinoma (ADC), and large cell carcinoma (LCC). SCC is still the most common histologic type of primary lung cancers in developing countries, although its ratio has decreased while that of adenocarcinoma increased over the years [6].

Selenium is an essential trace element for a lot of biologic processes and possesses anti-carcinogenic properties. Forty years ago, supplemental dietary selenium was found to play an important role in decreasing cancer risk [7]. Selenium deficiency in diet can increase incidence of cancers, including liver, prostate, lung, and colorectal cancers [8]. Its antitumor functions are mediated with selenium-binding protein 1 (SBP1, SELENBP1, hSP56) via binding selenium covalently [9, 10]. SBP1 can express abundantly in many normal human tissues [11, 12]. The expressions of SBP1 were reported to decrease markedly in numerous tumor types compared with their corresponding normal tissues. The expression reduction is associated with poor outcome in lung adenocarcinomas, breast cancer, gastric cancer, colorectal cancer, and renal cell carcinoma [13–17]. In our previous study, we had found that SBP1 was progressively decreased in the human bronchial epithelial carcinogenic processes and SBP1 expression could distinguish normal bronchial epithelium (NBE) from preneoplastic lesions and invasive LSCC. Knockdown of SBP1 in immortalized human bronchial epithelial cell line 16HBE cells significantly increased the efficiency of B[a]P-induced cell transformation [18]. However, there have been very few reports about the relationship between SBP1 expression and clinicopathological factors in LSCC. The specific role of SBP1 in prognosis of LSCC is still unknown. Therefore, in this study, we investigated the expression of SBP1 in LSCC and corresponding NBE tissues by immunohistochemistry and western blotting, evaluated the relationship of SBP1 expression and clincopathological factors, and further determined its prognostic significance via analyzing the correlation of SBP1 expression with survival.

## Methods

### Chemicals and antibodies

Anti-SBP1, β-actin monoclonal antibody, and violet-free methyl green were from Sigma–Aldrich. Horseradish peroxidase (HRP)-labeled secondary antibodies (goat anti-mouse IgG) and ECL detection reagent were purchased from Amersham Biosciences. Polyvinylidene difluoride (PVDF) membranes were from Millipore. Protease inhibitor was purchased from Roche Molecular Biochemicals. Coomassie (Bradford) Protein Assay Kit was obtained from Pierce. Standard solutions (bovine serum albumin) were from Merck Germany. SP kit and DAB developer were bought from Fuzhou Maixin.

### Patients and tissue specimen gender

Eighty-two patients with histologically confirmed LSCC were included in this study, all of whom were recruited from December 2007 to July 2008 at the Department of Cardiothoracic Surgery, The Second Affiliated Hospital of the University of South China. There were no age, gender, ethnicity, or tumor stage restrictions on patient enrolment. As variables possibly affect prognosis, we collected clinicopathological features including age, gender, smoking state, primary tumor (T) stage, TNM stage, regional lymph node metastasis, and distant metastasis determined according to the sixth edition of AJCC cancer staging manual [19]. All patients were selected at their first diagnosis and had not received chemotherapy, radiotherapy, and/or immunotherapy before pulmonary lobectomy. Every LSCC sample was matched with the corresponding normal bronchial epithelium tissues usually 5–10 cm away from the border of the main tumor lesions in the same patient. After surgeries, bronchi and tumor tissues were removed from the resected pulmonary lobes. Sixteen pairs of LSCC tissues and matched bronchi were stored at −80 °C for laser capture microdissection (LCM) and western blotting. Another 66 pairs were formalin-fixed and paraffin-embedded for immunohistochemistry. The diagnosis of primary LSCC and the corresponding NBE tissues was confirmed by two independent pathologists who were blinded to the original diagnoses. All of the survival status was regularly evaluated from the date of primary curative surgeries to July 31, 2013. Only the records of patients who had died of LSCC were considered as uncensored. Patients who died of a cause not related to LSCC and patients who were alive at the end of follow-up interval were recorded as censored. Every patient signed an informed consent form for the study which was approved by the local ethical committee. All clinical investigations were conducted according to the principles expressed in the Declaration of Helsinki.

### Laser capture microdissection

**Fig. 1:**
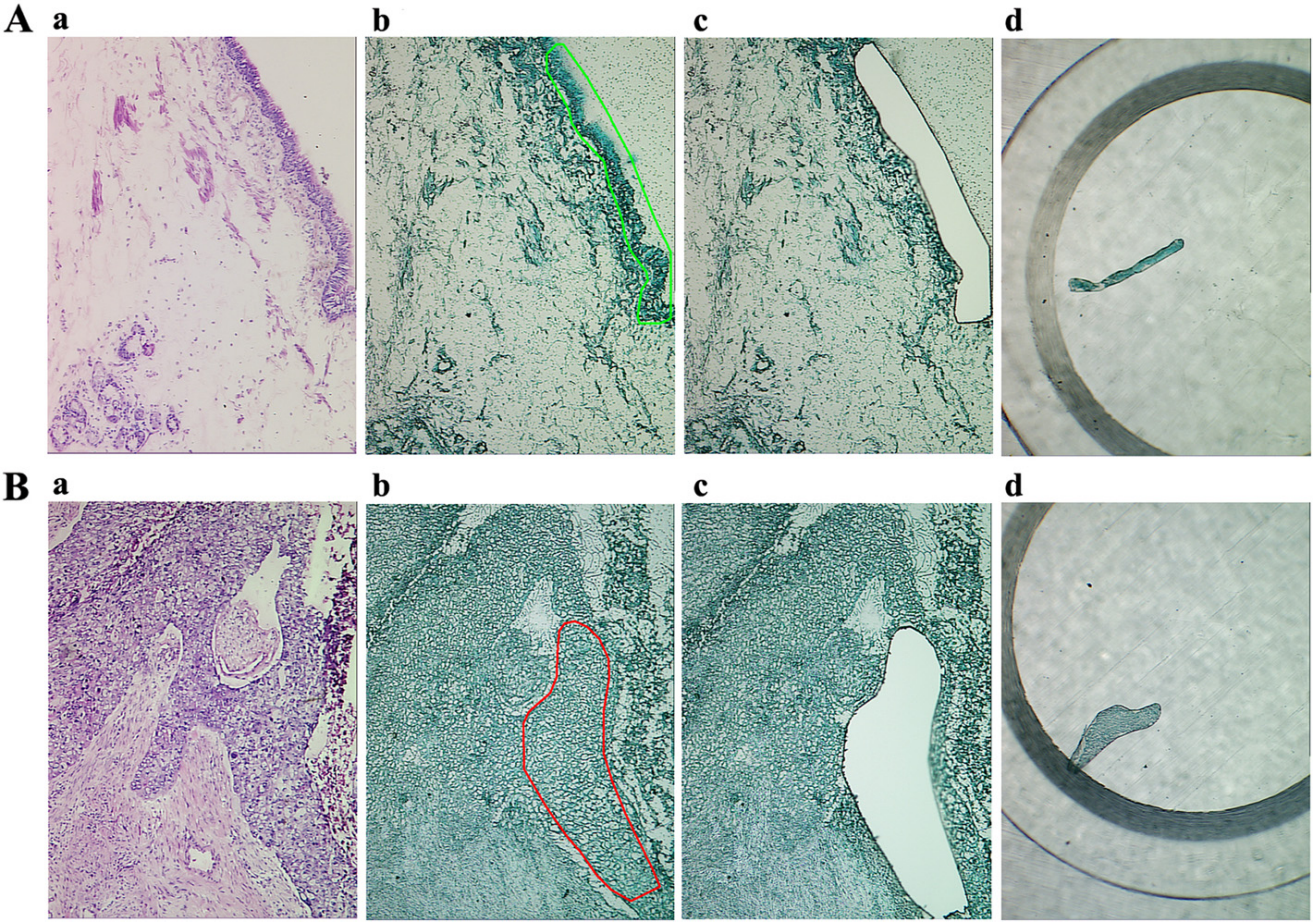
Purification of human normal bronchial epithelium and lung squamous cell carcinoma tissues by LCM. **a** H.E. staining of NBE (*a*), NBE before (*b*) and after (*c*) LCM, and captured NBE cells (*d*). **b** H.E. staining of LSCC (*a*), LSCC before (*b*) and after (*c*) LCM, and captured LSCC cells (*d*)

LCM was performed using a Leica AS LMD system (Leica) to purify the interest cells from LSCC tissues and matched NBE tissues as described previously [20]. Seven-micrometer-thick frozen sections of fresh LSCC and NBE were prepared using a Leica CM 1900 cryostat (Leica) at −25 °C. The sections were placed on membrane-coated glass slides (Leica), fixed in 75 % alcohol for 30 s, and stained with 0.5 % violet-free methyl green (Sigma). Following staining, all solutions for staining were supplemented with protease inhibitor cocktail tablets (Roche Molecular Biochemicals), the stained sections were air-dried and then subjected to LCM. Each cell population was determined to be 95 % homogeneous by microscopic visualization of the captured cells (Fig. 1). The microdissected cells were dissolved in lysis buffer (2 M thiourea, 7 M urea, 0.1 mM phenylmethylsulfonyl fluoride, 65 mM dithiothreitol) at 4 °C for 1 h and then centrifuged at 12,000 rpm for 30 min at 4 °C. The supernatant was transferred to a fresh tube and stored at −80 °C until western blotting.

### Measurement of tissue sample protein concentrations

The concentration of total proteins of tissue samples was decided according to Bradford assay method involving reacting the tissue samples with a dye that binds proteins. To measure the protein concentration, standard solutions (bovine serum albumin, Merck Germany) and tissue samples were prepared and Bradford reagent was added. The absorbance of tissue samples and standard solutions were measured at 595 nm after 10 min incubation at room temperature. A standard curve was prepared using the standard solution absorbance and the protein concentration of samples was estimated [21].

### Western blotting

Sixteen pairs of microdissected fresh LSCC and matched NBE tissues were used for western blotting as previously described by us [20]. In brief, 30 μg of lysates were separated by 10 % SDS-PAGE and transferred to PVDF membranes. Blots were incubated with primary anti-SBP1 antibody (1:500; Sigma) overnight at 4 °C, followed by incubation with a horseradish peroxidase-conjugated secondary antibody (1:3000; Amersham Biosciences) for 1 h at room temperature. The signal was visualized with ECL detection reagent (Amersham Biosciences) and quantitated by densitometry using ImageQuant image analysis system (Storm Optical Scanner, Molecular Dynamics). β-Actin was simultaneously detected using mouse anti-β-actin antibody (1:3000; Sigma) as a loading control.

### Immunohistochemistry and evaluation of staining

Immunohistochemistry was done on formalin-fixed and paraffin-embedded tissue specimens including 66 cases of LSCC and 66 cases of matched NBE. Briefly, 4 μm of tissue sections was deparaffinized, rehydrated, and treated with an antigen retrieval solution (10 mmol/l sodium citrate buffer, pH 6.0). The sections were incubated with anti-SBP1 (1:50; Sigma–Aldrich) antibody overnight at 4 °C and then were incubated with 1:1000 dilution of biotinylated secondary antibody. Immunoreactivity was visualized using 3′,3′-diaminobenzidine tetrachloride (DAB; Sigma–Aldrich) and counterstained with hematoxylin. In negative controls, primary antibodies were replaced by PBS.

Immunostaining was blindly evaluated by two investigators in an effort to provide a consensus on staining patterns under light microscopy. A quantitative score was performed by adding the score of staining intensity and the score of staining area for each case to assess the expression levels of the proteins as previously described by us [20]. At least 10 high-power fields were chosen randomly, and >1000 cells were counted for each section. First, a quantitative score was performed by estimating the percentage of immunopositive cells: 0, no staining of cells in any microscopic fields; 1+, <30 % of tissue stained positive; 2+, between 30 and 60 % stained positive; and 3+,>60 % stained positive. Second, the intensity of staining was scored by evaluating the average staining intensity of the positive cells (0, no staining; 1+, mild staining; 2+, moderate staining; 3+, intense staining). Finally, a total score (ranging 0~6) was obtained by adding the area score and the intensity score for each case. A combined staining score of ≤2 was considered to be low staining (negative expression); a score between 3 and 4 was considered to be moderate staining (expression); that between 5 and 6 was considered to be strong staining (high expression).

### Statistical analysis

All statistical analyses were performed using SPSS 15.0 software. The difference of SBP1 protein expressions between NBE and LSCC and the relationships between SBP1 expression and clinicopathological factors were analyzed using the *χ*^2^ test. Follow-up by telephone was carried out to obtain the information of patients’ outcomes. The follow-up period lasted up to 60 months. Overall survival was calculated from the time of surgery to the time of death. The deaths of the patients caused by LSCC were considered as outcomes; the deaths of the patients by other causes were censored, and the missing values were replaced by the series mean method. Overall survival curves were obtained using the Kaplan–Meier method, and log-rank testing was used to evaluate the statistically significant differences. Cox regression analysis was used to evaluate the prognostic significance of clinicopathological factors. *P* < 0.05 was considered as statistical significance.

## Results

### Expression of SBP1 in LSCC and NBE

**Fig. 2:**
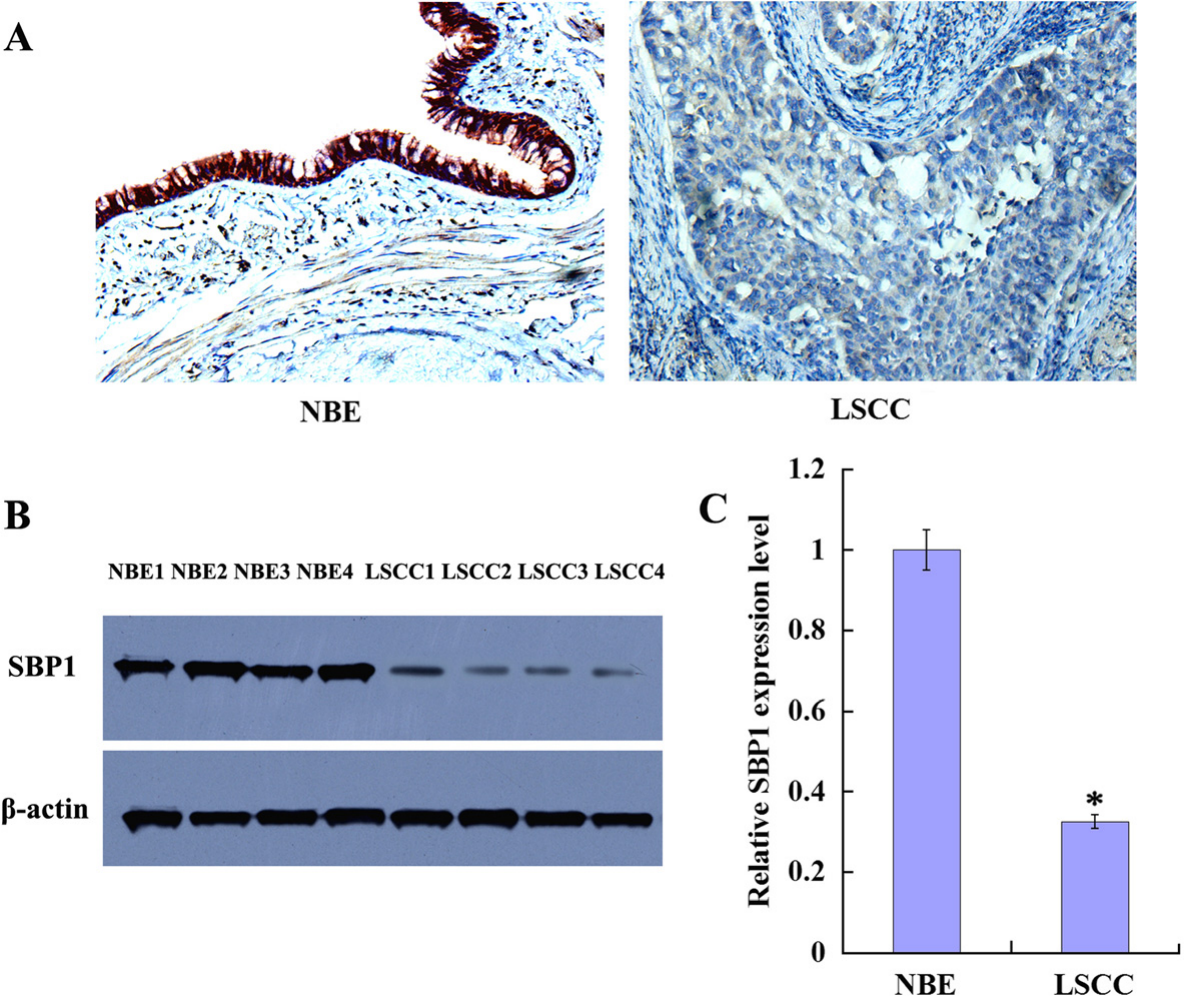
Expression of SBP1 in the human normal bronchial epithelium and lung squamous cell carcinoma tissues. **a** A representative result of immunohistochemistry shows expression of SBP1 is reduced in LSCC compared with the matched NBE. Original magnification, ×200. **b** A representative result of western blotting shows the expressions of SBP1 in the microdissected NBE and LSCC; **c** histogram shows the expression levels of SBP1 in NBE and LSCC tissues as determined by densitometric analysis. β-Actin is used as the internal loading control. *Columns*, mean from 16 cases of tissues; *bars*, SD (*P < 0.05 by one-way ANOVA)

SBP1 protein distribution was observed primarily in the cytoplasm and nucleus of cells (Fig. 2a). Immunohistochemical analysis demonstrated that SBP1 protein expression decreased significantly in LSCC compared to its abundance in the corresponding NBE. Among the 66 LSCC tissue samples, 63.6 % (42/66) stained were negative (low expression), only 36.4 % (24/66) stained were positive (diffuse cytoplasmic staining, moderate and high expression). However, there were 13.6 % (9/66) stained negative (low expression) and 86.4 % (57/66) stained positive (strong diffuse cytoplasmic staining and nuclear staining) among the 66 NBE tissue samples (Table 1, *P* < 0.05). The expressional levels of SBP1 protein were further verified by western blotting analysis, which were performed with 16 pairs of microdissected fresh LSCC and matched NBE tissues. Similarly, the expression of SBP1 protein was also found to be downregulated in all 16 human primary LSCC tissues compared with their matched NBE tissues (Fig. 2b, c). These results demonstrated that the expressional levels of SBP1 protein were markedly decreased in LSCC tissues compared with the corresponding NBE tissues.

**Table 1:** The difference of SBP1 expression between LSCC and normal bronchial epithelium

	*n*	Score			*P* value
		Low (0–2)	Moderate (3–4)	High (5–6)	
NBE	66	9	24	33	0.000*
LSCC	66	42	18	6	

*P < 0.05 by *χ* ^2^ test

### Correlation of SBP1 expression in LSCC with clinicopathologic factors

Table 2 showed the correlation of several clinicopathologic factors with SBP1 expression status among 66 cases of primary LSCC. The expression levels of SBP1 had not correlation with patients’ age, gender, smoking state, primary tumor stages (T), TNM clinical stages, and distant metastasis (M) (*P* > 0.05). However, SBP1 expressions were correlated with regional lymph node metastasis (N) (*P* < 0.05). These results might indicate that the reduction of SBP1 be associated with the progression of LSCC.

**Table 2:** Relationship between SBP1 expression and clinicopathological factors in lung squamous cell cancer

Variables	*n*	Score			*P* value
		Low (0–2)	Moderate (3–4)	High (5–6)	
Age, years					
<55	29	19	8	2	0.779
≥55	37	23	10	4	
Gender					
Male	34	22	9	3	0.852
Female	32	20	9	3	
Smoking					
Smoking	41	25	12	4	0.565
Non-smoking	25	17	6	2	
TNM clinical stage					
I–II	21	9	7	5	0.165
III–IV	45	33	11	1	
Primary tumor (T) stage					
T1–T2	37	27	7	3	0.075
T3–T4	29	15	11	3	
Regional lymph node metastasis (N)					
N0	29	14	10	5	0.022*
N1, N2, N3	37	28	8	1	
Distant metastasis (M)					
M0	52	30	16	6	0.053
M1	14	12	2	0	

*P < 0.05 by *χ* ^2^ test

### Correlation of SBP1 expression and survival of patients with LSCC

**Fig. 3:**
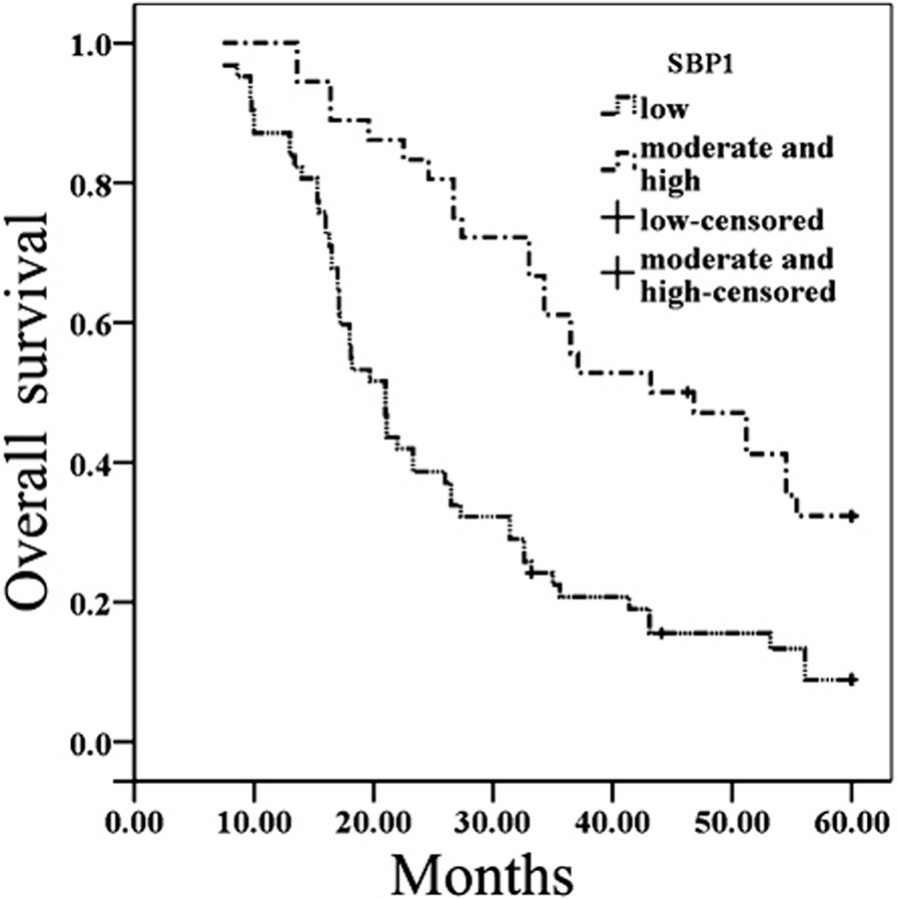
Kaplan–Meier survival plots for LSCC patients according to the expression levels of SBP1. SBP1 expression and overall survival (*P* = 0.000). *P* value was determined using a two-sided log-rank test

To verify whether the downregulation of SBP1 associated with the outcomes of patients with LSCC, we evaluated SBP1 as a prognostic factor among 66 patients with LSCC after surgical resection according to immunohistochemical SBP1 expressions. In the end of the study, 53 patients died, 10 patients were still alive, and 3 patients were lost during follow-up. The mean survival times of the patients with moderate and high expressions of SBP1 was 42.0 ± 16.2 months, which was higher than that of patients with low expression of SBP1 (26.1 ± 15.1 months, *P* < 0.01). The survival curves showed that the overall survival rate was significantly decreased with decreasing SBP1 expression (Fig. 3). To identify independent predictors for survival, univariate and multivariate Cox regression analyses were performed. Via univariate analysis, Table 3 showed that survival reduction was correlated with lymph node metastases, distant metastasis, advanced TNM stages, and decreasing SBP1 expression. Age and gender of patients, smoking state, and primary tumor stages (T) did not influence survival. The four significant prognostic factors determined by univariate analysis were included in a subsequent stepwise multivariate analysis. By multivariate analysis (Table 4), lymph node metastases, distant metastasis, advanced TNM stages, and decreasing SBP1 expression remained as the significantly independent prognostic factors for decreasing overall survival rate.

**Table 3:** Univariate Cox regression analysis of overall survivals

Variables	Overall survival	
	HR (95 % CI)	*P* value
Age (<55/≥55)	0.873 (0.558–1.365)	0.551
Gender (male/female)	1.078 (0.677–1.717)	0.752
Smoking (no/yes)	0.750 (0.481–1.171)	0.205
T stage (T1–T2/T3–T4)	1.339 (0.861–2.084)	0.196
TNM stage (I–II/III–IV)	6.060 (3.298–11.135)	0.000*
Tumor-node-metastasis (N/P)	11.021 (5.723–21.221)	0.000*
Distant metastasis (N/P)	5.813 (3.360–10.056)	0.000*
SBP1 expression (0–2/3–6)	0.385 (0.236–0.628)	0.000*

*HR* hazard ratio, *95 % CI* 95 % confidence interval, *N* negative, *P* positive

*P < 0.05

**Table 4:** Multivariate Cox regression analysis of overall survivals

Variables	Overall survival	
	HR (95 % CI)	*P* value
TNM stage (I–II/III–IV)	0.421 (0.182–0.973)	0.043*
Tumor-node-metastasis (N/P)	0.203 (0.087–0.470)	0.000*
Distant metastasis (N/P)	0.380 (0.213–0.677)	0.001*
SBP1 expression (0–2/3–6)	2.228 (1.329–3.737)	0.002*

*HR* hazard ratio, *95 % CI* 95 % confidence interval, *N* negative, *P* positive

*P < 0.05

## Discussion

Lung cancer is the most frequently occurring malignancy with increasing incidence, and it is also the leading cause of mortality in cancer-related deaths in China and worldwide [22, 23]. LSCC is the most common histological type of lung cancer. At present, the TNM staging system is considered as the most accurate predictor for LSCC [24]. Based on histopathology and extent of disease at presentation, the anatomic TNM staging system has reached its limit in providing critical information that may influence the strategies of treatments. However, pathologically similar tumors with comparable stages show a dramatically different response to the same therapy. Although surgery is the best therapeutic modality for patients with early stages of LSCC, the patients with the same pathological and clinical stages of LSCC display considerable variabilities in survival. Even after radical surgery, a significant proportion of patients may suffer from regional or distant recurrence. Therefore, there is an urgent need for finding new molecular markers that can distinguish between patients with unfavorable prognosis and others with better prognosis. If individuals with poor prognoses could be identified at the time of surgeries, their survival might be prolonged using more effective adjuvant therapies.

Selenium is an essential trace element involving antioxidative, antimutagenic, antiviral, and anticarcinogenic properties [25]. Some convincing epidemiological data showed there was a statistically significant inverse relationship between selenium levels and cancer risk [26–28]. It is suggested the anticancer action of selenium might be mediated by SBP1 as it is decreased in prostate cancer, colorectal cancer, and esophageal adenocarcinoma [29–31]. SBP1 displays tumor suppressor functions and plays a role in toxification/detoxification processes, cell growth regulation, cell motility, apoptosis, and intra-Golgi protein transport [32–35]. A research about lung adenocarcinoma showed SBP1 was significantly decreased in T2 to T4 stage tumors (versus T1 stage tumors) and bronchus-derived tumors (versus bronchioloalveolar adenocarcinoma) [13]. Hepatocellular carcinoma patients with lower SBP1 expression experienced shorter overall survival periods and higher rates of disease recurrence. SBP1 was reported as an independent risk factor for overall survival and disease recurrence [34]. In our previous studies, we found that knockdown of SBP1 in immortalized human bronchial epithelial cell line 16HBE cells significantly promoted cell proliferation, inhibited apoptosis, and increased the efficiency of B[a]P-induced cell transformation [18]. However, there was little information about the relationship of SBP1 expression and clinicopathological factors of LSCC. Meanwhile, the prognostic significance of SBP1 expression in LSCC is not yet to be clarified.

We examined SBP1 protein expression in LSCC tissues using western blotting and immunohistochemistry so as to investigate the role of SBP1 in LSCC because of the proteins as the cellular function molecules. Our results demonstrated that SBP1 was downregulated in LSCC compared with matched NBE tissues. The SBP1 proteins were detected both in the cytoplasm and nucleus, using immunohistochemical staining. In all, 36.4 % (24/66) of LSCC showed positive staining of SBP1. Our study showed that SBP1 expression was markedly diminished in lymph node metastasis (versus without lymph node metastasis) of patients by analyzing the correlation between SBP1 expression and clinicopathologic factors. The expression levels of SBP1 correlated with lymph node metastasis. The data revealed that the median survival time in patients with low-level expression of SBP1 appears shorter than that in patients with moderate and high SBP1 expressions. The median survival time of patients with SBP1 low-level expression was 26.1 ± 15.1 months, but for patients with moderate and high expression of SBP1, it was 42.0 ± 16.2 months. This is the first study for evaluating the prognostic value of SBP1 in LSCC patients. Survival analysis with Cox proportional hazard regression analysis and Kaplan–Meier method demonstrated that SBP1 expressions were closely related to the survival of LSCC, and reduced SBP1 expressions were an independently prognostic factor for poor overall survival in LSCC patients. Therefore, the data demonstrate that SBP1 expressions have the potential role for predicting the outcome of LSCC patients. The assessment of SBP1 expressions may, therefore, be used as an additional tool for identifying the patients at risk of tumor progression, and it may be a helpful criterion to optimize individual therapy management. Our findings have possible clinical applications.

## Conclusions

Our results indicate that SBP1 expression is reduced in LSCC and associated with lymph node metastasis of patients. Reduced SBP1 is an independent prognostic factor for poor overall survival in LSCC patients. SBP1 could serve as a potential prognostic marker for improving tumor classification of LSCC.

## Abbreviations

ADC: adenocarcinoma
IHC: immunohistochemistry
LCC: large cell carcinoma
LCM: laser capture microdissection
LSCC: lung squamous cell carcinoma
NBE: normal bronchial epithelium
NSCLC: non-small cell lung cancer
SBP1: selenium-binding protein 1
SCLC: small cell lung cancer
